# Prediction of Total Oral Intake by Videoendoscopic Evaluation in Patients With Acute Stroke

**DOI:** 10.7759/cureus.107474

**Published:** 2026-04-21

**Authors:** Ayaka Fujita, Manabu Iwata, Eiichi Tsuda

**Affiliations:** 1 Department of Rehabilitation Medicine, Hirosaki University Hospital, Hirosaki, JPN; 2 Department of Rehabilitation, Hirosaki Stroke and Rehabilitation Center, Hirosaki, JPN; 3 Department of Rehabilitation Medicine, Hirosaki University Graduate School of Medicine, Hirosaki, JPN

**Keywords:** acute stroke, dysphagia, flexible endoscopic evaluation of swallowing, hyodo-komagane score, oral diet outcome

## Abstract

Background: Post-stroke dysphagia is common and is linked to adverse clinical outcomes. The Hyodo-Komagane (H-K) score is an endoscopic index of dysphagia severity used during flexible endoscopic evaluation of swallowing (FEES) in Japan. However, its value for predicting oral intake outcome in acute stroke has not been fully established.

Objective: This study aimed to investigate whether the H-K score assessed during acute stroke is associated with oral diet (OD) at discharge after rehabilitation.

Methods: We retrospectively reviewed 137 patients with acute stroke who underwent FEES between September 2018 and March 2020 at the Hirosaki Stroke and Rehabilitation Center in Hirosaki, Japan. According to oral intake status at discharge, patients were assigned to either an OD group or a tube feeding (TF) group. Multivariable logistic regression was performed to identify factors independently associated with OD at discharge. Predictive ability was also evaluated using receiver operating characteristic analysis for age, National Institutes of Health Stroke Scale (NIHSS) score at admission, and H-K score.

Results: Of the 137 patients, 107 were classified into the OD group and 30 into the TF group. The OD group was younger and showed lower NIHSS and H-K scores than the TF group. In the multivariable analysis, age (odds ratio (OR), 0.94; 95% confidence interval (CI), 0.88-0.99; p = 0.02), NIHSS score at admission (OR, 0.93; 95% CI, 0.88-0.99; p = 0.02), and H-K score (OR, 0.56; 95% CI, 0.41-0.74; p < 0.0001) were independently associated with OD at discharge. The area under the curve was 0.655 for age, 0.666 for NIHSS score, and 0.810 for H-K score. The optimal cutoff value for the H-K score was 5, with 0.64 sensitivity and 0.84 specificity. A model combining age and H-K score showed an area under the curve of 0.835.

Conclusions: In patients with acute stroke and dysphagia who underwent FEES, the H-K score was associated with OD at discharge and showed the best predictive performance among the evaluated variables. Adding age provided only a small incremental improvement. The H-K score may be a useful bedside parameter for estimating OD outcome in acute stroke.

## Introduction

Dysphagia is a frequent and clinically important complication after stroke. Reported prevalence has varied widely across studies, ranging from 19% to 81% [1-3]. Although swallowing function may improve spontaneously or with treatment, dysphagia can persist in a substantial proportion of patients, and some symptoms remain even six months after stroke [4,5]. Post-stroke dysphagia is associated with aspiration pneumonia, longer hospitalization, and higher mortality [6,7]. Reduced oral intake may also lead to malnutrition and sarcopenia, both of which are linked to swallowing dysfunction [8,9]. For this reason, early prediction of oral intake outcome has clear clinical value in patients with stroke and dysphagia.

Several previous studies have investigated factors related to recovery of oral feeding or discontinuation of enteral tube feeding (TF) in patients with post-stroke dysphagia [10-17]. Reported predictors have included age [11,12,15,16], stroke severity [15,16], functional status [11,14,16,17], lesion characteristics [10,16], and aspiration-related findings [10-12]. However, the findings have not been consistent. Differences in patient selection, timing of assessment, outcome definitions, and swallowing assessment methods may partly explain these variations [5,13,14]. Therefore, in routine clinical practice, a simple and reliable method for estimating oral intake outcome during the acute phase is still needed.

Flexible endoscopic evaluation of swallowing (FEES) is widely used to assess swallowing function and aspiration risk in clinical settings. In Japan, the Hyodo-Komagane (H-K) score has been used as an endoscopic index of dysphagia severity during FEES [18,19]. The H-K score is based on four findings: salivary pooling, glottal closure or cough reflex induced by endoscopic stimulation, swallowing reflex initiation, and pharyngeal clearance after swallowing [18,19]. Because these findings reflect major aspects of swallowing physiology observed during endoscopy, the H-K score may be useful not only for grading dysphagia severity but also for estimating later recovery of oral intake.

Previous reports have suggested that Hyodo-based endoscopic scoring may be associated with aspiration risk during swallowing and with recovery of oral intake in patients with aspiration pneumonia [18,19]. However, evidence for the usefulness of the H-K score in predicting oral intake outcome, specifically in patients with stroke, remains limited. Most prognostic studies in stroke have focused on demographic factors, neurological severity, functional status, or tube-feeding outcomes rather than FEES-based composite scores [10-17]. As a result, it remains uncertain whether the H-K score assessed during acute stroke can independently predict total oral intake at discharge after rehabilitation.

In this study, we examined whether the H-K score measured during acute stroke was associated with total oral intake at discharge after rehabilitation. We also investigated whether combining the H-K score with other clinical variables could improve the prediction of oral intake outcome.

## Materials and methods

Patients

We retrospectively analyzed 137 consecutive patients with acute stroke who underwent FEES at the Hirosaki Stroke and Rehabilitation Center in Hirosaki, Japan, between September 2018 and March 2020. Acute stroke was defined as ischemic stroke or intracerebral hemorrhage diagnosed on the basis of clinical findings and routine neuroimaging, including computed tomography or magnetic resonance imaging.

At our center, FEES was not performed routinely in all patients with acute stroke. Instead, it was used selectively in patients with suspected dysphagia who were sufficiently alert to undergo endoscopic assessment. Specifically, FEES was performed in patients who either failed bedside swallowing screening by a speech-language-hearing therapist or were suspected of aspiration after oral intake had been initiated. Patients who died during hospitalization were excluded.

Clinical characteristics

Clinical data were collected retrospectively from the medical records. The assessed variables were sex, age at onset, National Institutes of Health Stroke Scale (NIHSS) score on admission [20], modified Rankin Scale (mRS) score [21] before stroke onset, and FEES-based swallowing findings. The FEES-based variables comprised the total H-K score and the following four subitems: salivary pooling, cough reflex, swallowing reflex initiation, and pharyngeal clearance [18,19]. There were no missing data for the variables included in the final analysis.

Ethics

This study received approval from the ethics committee of the Hirosaki Stroke and Rehabilitation Center (protocol number 23A001) and was conducted in accordance with the Declaration of Helsinki. Given the retrospective nature of the study, written informed consent was waived, and an opt-out approach was used.

Stroke rehabilitation

All patients underwent stroke rehabilitation within 24 hours of admission. Rehabilitation was delivered by a multidisciplinary stroke team that included physicians and allied health professionals, such as nurses, physical therapists, occupational therapists, speech-language-hearing therapists, pharmacists, and nutritionists.

Hyodo-Komagane score

In Japan, the H-K score is commonly applied during FEES as an index of dysphagia severity [18]. The score includes four endoscopic findings. These comprise the presence of secretions accumulating in the vallecula and piriform sinuses, induction of the glottal closure reflex by contact of the endoscope with the epiglottis or arytenoid, initiation of the swallowing reflex, and pharyngeal clearance after swallowing dyed water [18].

Statistical analysis

Patients were divided into two groups according to oral intake status at discharge: an oral diet (OD) group and a tube feeding (TF) group. In this study, OD at discharge was defined as total oral intake without dependence on tube feeding. Continuous and ordinal variables are presented as medians with interquartile ranges, whereas categorical variables are expressed as numbers and percentages. Differences between groups were assessed with the Wilcoxon rank-sum test for continuous or ordinal data, whereas categorical variables were analyzed using the chi-square test or Fisher’s exact test.

The predictive performance of age, NIHSS score at admission, and H-K score for OD status at discharge was evaluated using receiver operating characteristic (ROC) curve analysis. Optimal cutoff values were identified from the ROC curves, and sensitivity and specificity were calculated.

We then used multivariable logistic regression to examine factors independently associated with OD at discharge. Age, NIHSS score at admission, and H-K score were entered into the model because they were considered clinically relevant predictors of OD outcome based on prior literature and the study objective. Given the sample size and number of outcome events, the number of predictors in the final model was kept limited to reduce the risk of overfitting. Results are reported as odds ratios (ORs) with 95% confidence intervals (CIs). All analyses were two-sided, and statistical significance was defined as a P value < 0.05. Statistical analyses were conducted using JMP Student Edition version 18.2.0 (SAS Institute Inc., Cary, NC, USA).

## Results

Univariate analysis

The baseline characteristics are summarized in Table 1.

**Table 1:** Baseline characteristics of patients according to oral diet status at discharge Values are presented as n (%) or median (IQR). Comparisons between groups were performed using the Mann–Whitney U test for continuous variables and the chi-square test for categorical variables. Z values represent standardized test statistics for the Mann–Whitney U test. The overall mRS distribution was compared using Fisher’s exact test; no individual statistical test was performed for mRS subcategories. OD: oral diet, TF: tube feeding, mRS: modified Rankin Scale; NIHSS: National Institutes of Health Stroke Scale, H-K: Hyodo-Komagane

Variables	OD group (n = 107)		TF group (n = 30)	Test statistic	P-value
Male sex, n (%)	67 (62.6)	15 (50.0)		χ² = 1.55	0.22
Age, years	79 (70–84)	83.5 (77–88.0)		Z = 2.58	0.005*
mRS before stroke onset	N/A	N/A		Fisher’s exact test	0.47
mRS 0	51 (47.7)	14 (46)		N/A	N/A
mRS 1	20 (18.7)	4 (13.3)		N/A	N/A
mRS 2	12 (11.2)	1 (3.1)		N/A	N/A
mRS 3	12 (11.2)	4 (12.5)		N/A	N/A
mRS 4	8 (7.5)	5 (16.7)		N/A	N/A
mRS 5	4 (3.7)	2 (6.7)		N/A	N/A
NIHSS on admission	11 (5–17)	14.5 (10–23.3)		Z = 2.77	0.006*
H-K score	5 (4–6)	7 (6–8)		Z = 5.24	<0.0001*
Pooling	1 (1–1)	2 (1–3)		Z = 3.16	0.0016*
Cough	1 (1–1)	1 (1–2)		Z = 1.00	0.32
Swallow	2 (1–2)	2 (2–3)		Z = 4.23	<0.0001*
Clearance	1 (0–1)	1 (1–2)		Z = 3.80	<0.0001*

The study cohort comprised 137 patients, of whom 107 were in the OD group and 30 were in the TF group. The OD group was younger than the TF group (79 (70-84) vs. 83.5 (77-88.0) years, P = 0.005). Sex (62.6% vs. 50.0%, P = 0.21) and pre-stroke mRS score (P = 0.46) did not differ significantly between the groups.

By contrast, the OD group showed lower NIHSS scores at admission (11 (5-17) vs. 14.5 (10-23.3), P = 0.006) and lower total H-K scores (5 (4-6) vs. 7 (6-8), P < 0.0001).

Among the H-K score subitems, pooling (P = 0.0016), swallowing reflex initiation (P < 0.0001), and pharyngeal clearance (P < 0.0001) differed between groups, whereas no difference was observed for cough reflex (P = 0.310).

Multivariable logistic regression model

Factors associated with OD at discharge were examined using multivariable logistic regression. Age (odds ratio (OR), 0.94; 95% confidence interval (CI), 0.88-0.99; P = 0.02), NIHSS score at admission (OR, 0.93; 95% CI, 0.88-0.99; P = 0.02), and H-K score (OR, 0.56; 95% CI, 0.41-0.74; P < 0.0001) were independently associated with OD at discharge (Table 2).

**Table 2:** Multivariable logistic regression analysis for oral diet at discharge NIHSS: National Institutes of Health Stroke Scale, H-K score: Hyodo-Komagane Score

	Odds ratio	95% confidence Interval	P-value
Age, years	0.94	0.88–0.99	0.02*
NIHSS on admission	0.93	0.88–0.99	0.02*
H-K score	0.56	0.41–0.74	<0.0001*

ROC curve analysis

**Figure 1:**
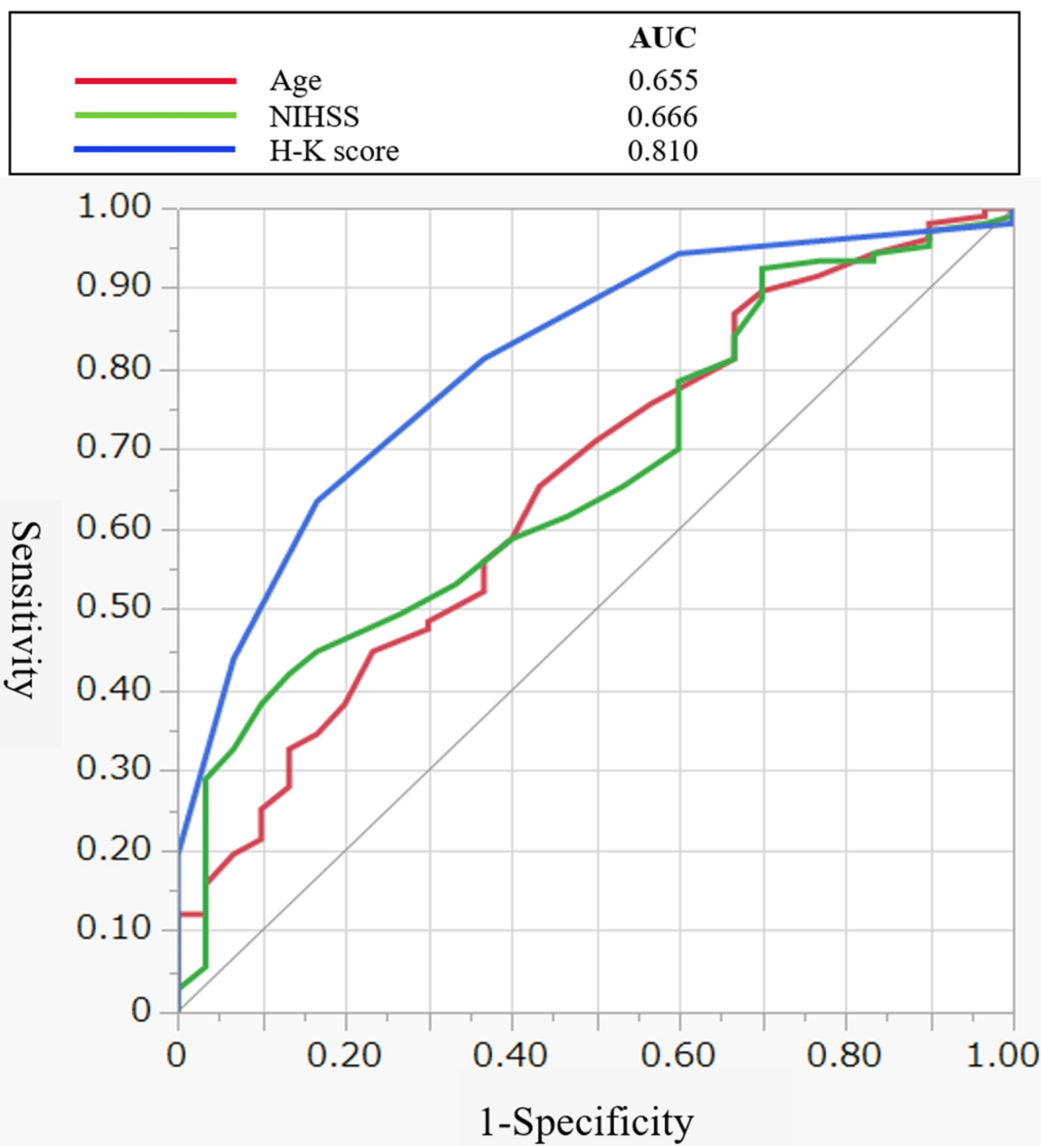
Receiver operating characteristic (ROC) curves for predicting oral intake at discharge. The blue line represents the Hyodo–Komagane (H-K) score, the green line represents the National Institutes of Health Stroke Scale (NIHSS) score, and the red line represents age. The area under the curve (AUC) was 0.810 (95% CI: 0.728–0.891) for the H-K score, 0.666 (95% CI: 0.561–0.77) for NIHSS, and 0.655 (95% CI: 0.544–0.765) for age. Sensitivity is plotted against 1 − specificity.

The predictive performance of age, NIHSS score at admission, and H-K score for OD at discharge was evaluated using ROC curve analysis. The AUC values were 0.655 (95% CI: 0.544-0.765) for age, 0.666 (95% CI: 0.561-0.77) for the NIHSS score, and 0.810 (95% CI: 0.728-0.891) for the H-K score (Figure 1). The optimal cutoff values were 82 years for age, 8 for the NIHSS score, and 5 for the H-K score.

The optimal cutoff values were 82 years for age, 8 for the NIHSS score, and 5 for the H-K score.

**Figure 2:**
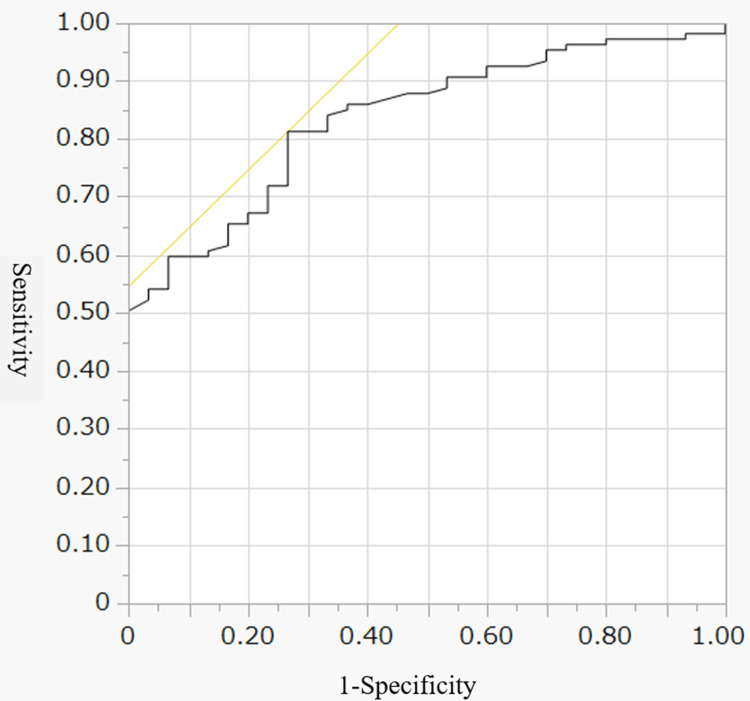
ROC curve for the combination model including age and H-K score in predicting oral diet at discharge after stroke (AUC = 0.835; 95% CI: 0.765–0.906). The model showed improved discriminative ability compared with individual variables. AUC: area under the curve; H-K score: Hyodo-Komagane score

Among these variables, the H-K score provided the highest discrimination, with a sensitivity of 0.64 and a specificity of 0.84. A combined model including age and the H-K score showed a slightly higher AUC of 0.835 (95% CI: 0.765-0.906) (Figure 2).

## Discussion

This study examined predictors of OD at discharge in patients with acute stroke who underwent FEES. The main finding was that the H-K score was independently associated with OD at discharge. In addition, age and NIHSS score at admission were also independently associated with the outcome. Among the evaluated variables, the H-K score showed the highest discriminative ability, with an AUC of 0.810. A combination model including age and the H-K score showed a slightly higher AUC of 0.835. These findings suggest that the H-K score is a practical FEES-based parameter for estimating OD outcome in this population.

Previous studies have reported that age, stroke severity, functional status, lesion characteristics, and aspiration-related findings are associated with recovery of oral intake after stroke [10-17]. In the present study, age and NIHSS score were also independently associated with OD outcome, which is consistent with earlier reports showing that older age and greater stroke severity are linked to poorer swallowing recovery [11,12,15-17]. Importantly, the H-K score showed better predictive performance than age or NIHSS score alone. This suggests that direct endoscopic assessment captures clinically relevant aspects of swallowing function that are not fully reflected by demographic characteristics or overall neurological severity. Because the H-K score incorporates multiple physiological components of swallowing observed during FEES, it may better represent the integrated functional status of swallowing at the time of assessment. The observed associations may also have been influenced by institutional factors, such as local rehabilitation practices and clinical decision-making regarding oral intake. Therefore, these factors should be taken into account when interpreting the results.

The H-K score reflects multiple components of swallowing physiology observed during FEES, including salivary pooling, reflex elicitation, swallowing reflex initiation, and pharyngeal clearance [18,19]. The total H-K score differed significantly between the two groups, with lower values observed in the OD group. In addition, three subitems, pooling, swallowing reflex initiation, and pharyngeal clearance, differed significantly between groups, whereas the cough reflex did not. These findings suggest that impaired secretion management, delayed swallow initiation, and reduced pharyngeal clearance may be more closely related to OD outcome than reflex elicitation alone. Previous studies have also reported that Hyodo-based scoring is useful for predicting aspiration risk and recovery of oral intake in other dysphagia populations, including aspiration pneumonia [18,19]. Our results extend these observations to patients with acute stroke.

From a clinical perspective, the H-K score may be useful because it is a simple bedside tool directly linked to swallowing function. In this study, the cutoff value of the H-K score for predicting OD at discharge was 5, with a sensitivity of 0.64 and a specificity of 0.84. Clinically, the cutoff value of 5 may serve as a practical reference point for estimating OD outcome at discharge, although it should be interpreted together with the individual FEES findings because the H-K score is a composite index. This level of specificity may help identify patients who are likely to remain dependent on TF and who may require closer nutritional management, swallowing rehabilitation, and discharge planning. Although the combination model, including age, slightly improved the AUC, the additional benefit was modest, suggesting that the H-K score alone captures much of the relevant predictive information.

Several limitations should be considered. This study was conducted retrospectively at a single center, and the possibility of selection bias remains. FEES was not performed routinely in all patients with acute stroke but was selectively performed in patients with suspected dysphagia who were sufficiently alert to undergo endoscopic assessment. Therefore, patients with very mild dysphagia and those with severe conditions who could not tolerate the procedure may have been underrepresented. Second, the timing of FEES was not standardized during the acute phase, which may have influenced the H-K scores. Third, model calibration was not assessed, and external validation was not performed. Therefore, the generalizability and predictive performance of the model should be interpreted with caution. In addition, several potential confounders were not included in the final model, such as comorbidities, nutritional status, lesion characteristics, and the timing of FEES. These variables were not consistently available in the retrospective dataset and, therefore, could not be reliably incorporated. Furthermore, to minimize the risk of overfitting given the sample size and number of outcome events, we intentionally limited the number of predictors in the multivariable model. As a result, residual confounding cannot be excluded, and the findings should be interpreted with caution.

Finally, OD at discharge was used as the outcome measure. Although clinically relevant, this outcome may have been influenced by institutional practice patterns and discharge timing. Therefore, the findings should be interpreted with caution and validated in prospective multicenter studies.

## Conclusions

The H-K score was independently associated with OD at discharge in patients with acute stroke and dysphagia who underwent FEES. Among the evaluated variables, the H-K score showed the highest predictive ability, and the combination model including age and the H-K score provided a small additional improvement. These findings suggest that the H-K score may be a useful bedside parameter for estimating OD outcome and informing nutritional management, swallowing rehabilitation, and discharge planning during the acute phase.
